# Development and validation of a rapid visual technique for left ventricular hypertrophy detection from the electrocardiogram

**DOI:** 10.3389/fcvm.2023.1251511

**Published:** 2023-08-23

**Authors:** Sulaiman Somani, J. Weston Hughes, Euan A. Ashley, Ronald M. Witteles, Marco V. Perez

**Affiliations:** ^1^ Division of Internal Medicine, Department of Medicine, Stanford University, Stanford, CA, United States; ^2^ Stanford Cardiovascular Institute, Stanford, CA, United States; ^3^ Division of Cardiovascular Medicine, Department of Medicine, Stanford University, Stanford, CA, United States; ^4^ Department of Computer Science, Stanford University, Stanford, CA, United States

**Keywords:** ECG, LVH, LVH detection, echo, left ventricular hypertrophy

## Abstract

**Introduction:**

Left ventricular hypertrophy (LVH) detection techniques on by electrocardiogram (ECG) are cumbersome to remember with modest performance. This study validated a rapid technique for LVH detection and measured its performance against other techniques.

**Methods:**

This was a retrospective cohort study of patients at Stanford Health Care who received ECGs and resting transthoracic echocardiograms (TTE) from 2006 through 2018. The novel technique, Witteles-Somani (WS), assesses for S- and R-wave overlap on adjacent precordial leads. The WS, Sokolow-Lyon, Cornell, and Peguero-Lo Presti techniques were algorithmically implemented on ECGs. Classification metrics, receiver-operator curves, and Pearson correlations measured performance. Age- and sex-adjusted Cox proportional hazard models evaluated associations between incident cardiovascular outcomes and each technique.

**Results:**

A total of 53,333 ECG-TTE pairs from 18,873 patients were identified. Of all ECG-TTE pairs, 21,638 (40.6%) had TTE-diagnosed LVH. The WS technique had a sensitivity of 0.46, specificity of 0.66, and AUROC of 0.56, compared to Sokolow-Lyon (AUROC 0.55), Cornell (AUROC 0.63), and Peguero-Lo Presti (AUROC 0.63). Patients meeting LVH by WS technique had a higher risk of cardiovascular mortality [HR 1.18, 95% CI (1.12, 1.24), *P* < 0.001] and a higher risk of developing any cardiovascular disease [HR 1.29, 95% CI (1.22, 1.36), *P* < 0.001], myocardial infarction [HR 1.60, 95% CI (1.44, 1.78), *P* < 0.005], and heart failure [HR 1.24, 95% CI (1.17, 1.32), *P* < 0.001].

**Conclusions:**

The WS criteria is a rapid visual technique for LVH detection with performance like other LVH detection techniques and is associated with incident cardiovascular outcomes.

## Introduction

Left ventricular (LV) hypertrophy (LVH) is the presence of increased LV mass and is most commonly driven by a pathologic response to chronic afterload stressors (e.g., hypertension, aortic valve disease) or inherited myopathies (e.g., hypertrophic cardiomyopathy) (1). It has been correlated with all-cause and cardiovascular mortality, as well as the incidence of other cardiovascular diseases (e.g., atrial fibrillation, heart failure), suggesting a possible role for earlier intervention (2). Existing techniques for LVH detection on electrocardiograms (ECG) are insensitive and can be laborious to remember and implement at the bedside (3). Artificial intelligence (AI) models have shown promise in improving screening (4), but suffer from deployment issues given the infancy of healthcare implementation science and infrastructure limitations to expand to resource-limited care centers, especially in primary care (5). We hypothesized that high early precordial lead (directed away from the LV) S-wave amplitudes and high later precordial lead (directed towards the LV) R-wave amplitudes would suggest a high or delayed LV depolarization signal, and that overlap of these on adjacent precordial leads would be associated with LVH. We therefore developed a fast and simple technique for LVH detection by ECG based on any QRS complex overlap on the precordial leads, validated its performance against existing techniques in a large validation cohort, and evaluated its association with the incidence of future cardiovascular events.

## Methods

**Figure 1:**
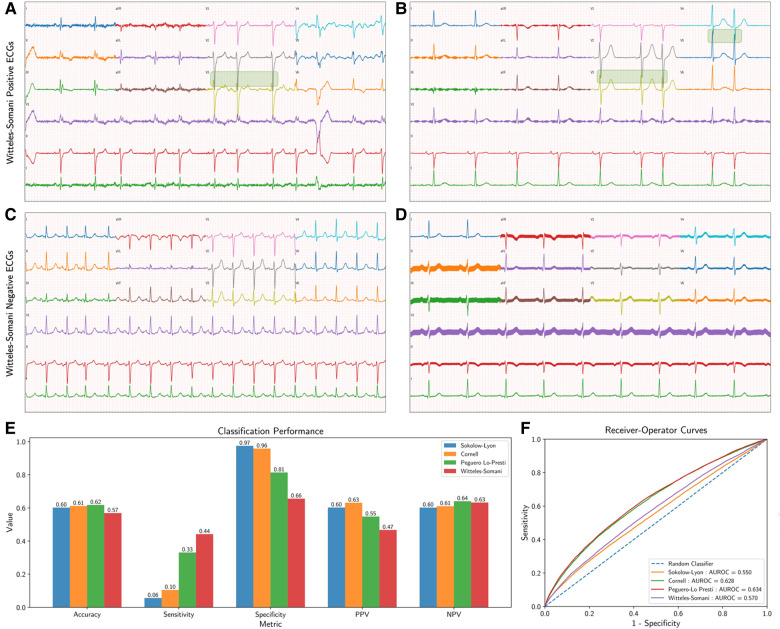
Witteles–Somani technique and performance. This figure demonstrates two examples of WS-positive (**A,B**) and WS-negative ECGs (**C,D**); areas of precordial overlap contributing to the positive diagnosis are overlaid with a green box. Performance measures for the various LVH detection techniques are also shown; performance on simple classification of LVH at their pre-specified thresholds (**E**) and changes in sensitivity and specificity as demonstrated by receiver-operator curves at various thresholds (**F**). LVH, left ventricular hypertrophy; WS, Witteles–Somani technique.

The Witteles–Somani (WS) criteria is defined here as the overlap of QRS complexes between adjacent precordial leads, which can easily be detected visually (Figures 1A–D). The criteria were automated in this analysis as the sum of the height of the S-wave from lead V_i_ and height of the R-wave from lead V_i + 1_ exceeding 20 mm, which is equal to 2 mV on conventional 12-lead ECG displays calibrated at 10 mm per 1 mV.

We conducted a retrospective cohort analysis that included patients ≥18 years of age at Stanford Health Care who had a resting transthoracic echocardiogram (TTE) and a 12-lead resting ECG performed between March 1, 2006 and May 31, 2018. ECGs closest in time to and within one year of each TTE were used to create a unique ECG-TTE pair. ECGs with bundle branch blocks (left and right), paced rhythms, or quality control warnings were excluded from this study. Demographic data and medical history were collected from the electronic health record. Cardiovascular mortality and incident cardiovascular diseases including heart failure and myocardial infarction were collected from diagnosis codes (Supplementary Table S1) in the medical record.

LV mass index (LVMI) for each echocardiogram was calculated using the Devereaux formula for LVH and normalized to the body surface area (BSA); LVH was defined as LVMI >95 g/m^2^ in males and LVMI >115 g/m^2^ in females (6). This study was approved by the Stanford University Institutional Review Board (IRB-41045). Full details on data acquisition, representation, and exclusion criteria are discussed in the Supplementary Methods.

To compare the WS criteria with other described methods of ECG-based LVH detection, we algorithmically implemented the Sokolow–Lyon, Cornell, and Peguero–Lo Presti techniques for evaluation from these ECGs (7–9). Because all LVH detection techniques rely on R- and S-wave amplitudes, QRS complexes from raw ECG waveforms were identified using the Christov algorithm (10). Additional details on implementation are discussed in the Supplementary Methods.

We generated point estimates for classification metrics—accuracy, sensitivity, specificity, positive predictive value (PPV), negative predictive value (NPV), and odds ratios. The McNemar test was used to assess agreement for LVH detected by each technique with LVH detected by TTE (null hypothesis). We performed sensitivity analyses to determine how sex-based threshold adjustment and incorporation of other visual ECG signs previously linked to etiologies of HCM (e.g., ST-segment depressions, left atrial enlargement, and T-wave inversions) could affect the performance of the WS technique (11). We generated receiver-operator curves (ROC) and their areas (AUROC) by using R- and S-wave measurements from each technique (maximum across the median of all criterion values across all heartbeats, MMCV; see Supplementary Methods) to assess threshold-independent performance. Next, we calculated the Pearson statistic to assess the linear correlation between the MMCV for each ECG and the LVMI. Finally, we developed age- and sex-adjusted Cox proportional hazards models for each technique and TTE-based LVH to determine the hazard ratios for cardiovascular mortality and incident myocardial infarction, heart failure, and any cardiovascular disease (defined as the incidence of MI, stroke/hemorrhage, sudden cardiac death, and heart failure hospitalization). Additional details are discussed in the Supplementary Methods.

## Results

A total of 53,333 ECG-TTE pairs from 18,873 patients were included in the analysis. In this cohort, the average age was 67.1 years ± 16.1 years and 49.8% were female, 6.1% were Black, 13.3% were Asian, and 7% were Hispanic. Of all ECG-TTE pairs, 21,638 (40.6%) had TTE-diagnosed LVH.

On univariate analysis, patients who were positive by the WS technique were more likely to be male (65.9% vs. 45.1%, *P* < 0.001) and be dialysis dependent (6.2% vs. 5.2%, *P* < 0.001), but less likely to have hypertension (45.6% vs. 47.7%, *P* < 0.001), diabetes (13.4% vs. 15.8%, *P* < 0.001), and prior MI (8.3% vs. 9.5%, *P* < 0.001). There were no statistical differences in age and history of heart failure, coronary artery disease, stroke, or ASCVD between the WS-positive and WS-negative cohorts (Table 1).

**Table 1:** Characteristics of patients positive or negative by the Witteles–Somani technique.

	WS Negative	WS Positive	*P*-Value
*N* (ECG-TTE Pairs)	32,864	20,469	
Age Median [Q1,Q3]	68.1 [57.2,77.0]	67.9 [56.1,77.9]	0.139
Female *N* (%)	18,035 (54.9)	6,985 (34.1)	<0.001
Hispanic or Latino *N* (%)	2,437 (7.4)	1,520 (7.4)	0.978
Race *N* (%)			
American Indian or Alaska Native	87 (0.3)	102 (0.5)	
Asian	3,230 (9.8)	3,220 (15.7)	
Black or African American	1,530 (4.7)	1,308 (6.4)	<0.001
Native Hawaiian or Other Pacific Islander	279 (0.8)	217 (1.1)	
Unknown	4,936 (15.0)	3,423 (16.7)	
White	22,802 (69.4)	12,199 (59.6)	
Hypertension *N* (%)	15,677 (47.7)	9,333 (45.6)	<0.001
Diabetes *N* (%)	5,207 (15.8)	2,745 (13.4)	<0.001
CKD stage *N* (%)			
1	178 (0.5)	197 (1.0)	<0.001
2	1,438 (4.4)	885 (4.3)	
3	3,180 (9.7)	1,932 (9.4)	
4	1,130 (3.4)	631 (3.1)	
5	668 (2.0)	594 (2.9)	
None	24,867 (75.7)	15,425 (75.4)	
Unstaged	1,403 (4.3)	805 (3.9)	
Dialysis-dependence *N* (%)	1,710 (5.2)	1,262 (6.2)	<0.001
Heart failure *N* (%)	5,786 (17.6)	3,715 (18.1)	0.113
Coronary artery disease *N* (%)	3,630 (11.0)	2,300 (11.2)	0.504
History of myocardial infarction *N* (%)	3,123 (9.5)	1,708 (8.3)	<0.001
History of stroke *N* (%)	1,661 (5.1)	1,084 (5.3)	0.227
Atherosclerotic cardiovascular disease *N* (%)	4,414 (13.4)	2,644 (12.9)	0.091

The WS technique had a sensitivity of 0.44, a specificity of 0.66, and an accuracy of 0.57 for detecting LVH. Other performance measures of WS and other LVH criteria are shown in Figure 1E and Supplementary Table S3. All techniques—Sokolow–Lyon (*χ*^2^ = 18148.4, *P* < 0.001), Cornell (*χ*^2^ = 15725.6, *P* < 0.001), Peguero–Lo Presti (*χ*^2^ = 3603.6, *P* < 0.001), and WS (*χ*^2^ = 59.3, *P* < 0.001), rejected the null hypothesis of statistical similarity between technique-based LVH and TTE-based LVH. The WS technique had an AUROC 0.56 for classifying LVH independent of pre-established thresholds, compared to Sokolow–Lyon (AUROC 0.55), Cornell (AUROC 0.63), and Peguero–Lo Presti (AUROC 0.63) (Figure 1F). Adjusting the threshold for LVH detection based on patient sex by 10 mm (2 “big” boxes on traditional 12-lead ECGs) increased the performance of the WS technique (AUROC 0.60, sensitivity 0.75, specificity 0.37), as shown in Supplementary Figure S2. Model performance from combining various techniques together and from inclusion of other visual ECG criteria is shown in Supplementary Tables S4, S5, respectively. Correlations of each technique with LVMI are shown in Supplementary Figure S3.

**Figure 2:**
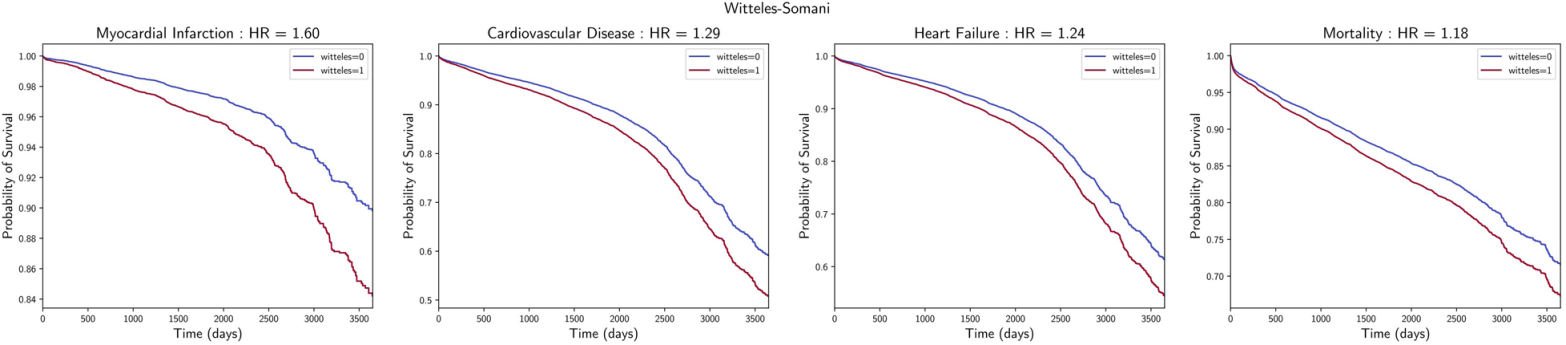
Association with cardiovascular outcomes. Survival curves of incidental myocardial infarction, cardiovascular disease, heart failure, and mortality, with associated hazard ratios and *P*-values for those with and without LVH, as detected by the WS technique.

WS-positive ECG-TTE pairs had a higher risk of cardiovascular mortality [HR 1.18, 95% CI (1.12, 1.24), *P* < 0.001] and a higher risk of developing any cardiovascular disease [HR 1.29, 95% CI (1.22, 1.36), *P* < 0.001], myocardial infarction [HR 1.60, 95% CI (1.44, 1.78), *P* < 0.005], and heart failure [HR 1.24, 95% CI (1.17, 1.32), *P* < 0.001] (Figure 2). These associations remained statistically significant when using the MMCV instead (Supplementary Figure S4). Analyses for the other LVH detection techniques were also performed, demonstrating that these, except Sokolow–Lyon, were also associated with higher risk of mortality and development of CVD, MI, and HF to similar degrees as the WS technique (Supplementary Figures S5–S8 and Supplementary Table S6).

## Discussion

In this brief report, we describe a novel technique for LVH detection from the ECG which can be performed with rapid visual assessment. We evaluate the performance of this technique, along with those of the Sokolow–Lyon, Cornell, and Peguero–Lo Presti techniques, on a large ECG-TTE linked dataset. We find that the WS technique has similar test characteristics as other commonly employed ECG-based techniques and is simpler to remember and implement. In addition, the WS technique criteria had a statistically significant correlation with LVMI and is associated with 10-year cardiovascular mortality and development of myocardial infarction, heart failure, and any cardiovascular disease.

Our work leverages a large institutional dataset to improve assessment of established LVH criteria and our novel WS criteria. The dataset in this study, composed of over 123,000 ECGs and 35,000 TTEs across over 30,000 patients, is much larger than the datasets used in other primary validation studies of LVH criteria (3). In our dataset, we found lower sensitivities for the three established LVH techniques than those reported in literature elsewhere. By nature of its larger size, our dataset may have captured a broader spectra of disease manifestations, such as varying degrees of hypertensive heart disease, and potentially other confounders of elevated LV mass on TTE but without LVH, such as infiltrative myocardial diseases (e.g., amyloidosis). The decreased incidence of hypertension in the WS-positive group may reflect capture of individuals with high voltages without the presence of LVH, as seen in young athletes for whom lateral ST-depressions and T-wave inversions are stronger discriminatory features (11). While sensitivity analyses on the WS technique failed to show an improvement in performance by incorporating these repolarization features, adjustment for female sex modestly increased the overall performance, at the cost of increasing the technique's complexity.

The WS technique is unique in that it relies on either high R- and S-voltages between adjacent leads, which favors high-amplitude isoelectric signals that likely reflect myocardial hypertrophy (barring longer chest-wall to myocardial distances, which may be present in obese patients or those with obstructive lung disease). Other techniques, by focusing on R and S waves in non-adjacent leads, can embed other contextual information. For example, a prominent S-wave in V4, a critical component of the Peguero–Lo Presti technique, may reflect delayed R-wave progression, the differential for which includes LVH and HCM.

Effective screening techniques for LVH require a highly sensitive, moderately specific, and low-cost solution and remain an active area of research. The WS technique detects LVH more sensitively at the cost of lower specificity, both of which are further enhanced when adjusting for sex (Supplementary Figure S2). Combinatorial evaluation of all LVH techniques did not reveal one combination that maximized sensitivity without a corresponding drop in specificity (Supplementary Table S4). Incorporating other diagnostic ECG findings known to correlate with LVH, such as left atrial enlargement, ST-depressions, and T-wave inversions, increased the total specificity, but with a significant drop in sensitivity (Supplementary Table S5). While beyond the scope of this present work, future work may investigate the impact of combining these criteria with elements from clinical history (e.g., demographics, comorbidities), physical exam findings (e.g., S4 on cardiac exam), and laboratory findings (e.g., brain natriuretic peptide, troponin) on LVH screening, as well as the cost effectiveness and clinical impact of a LVH screening pipeline. Ultimately, the performance for LVH detection across all techniques was modest. Artificial intelligence techniques like deep learning have demonstrated the potential to improve LVH detection on ECG, which far outperforms these criteria (12, 13). However, until these models can be made widely accessible to use or become naturally embedded in pre-existing ECG machines, manual techniques like the ones in this manuscript remain the likely most commonly utilized option for evaluating LVH from an ECG.

Ultimately, LVH has a long history of carrying prognostic value, which continues to fuel the development of techniques to increase its detection. Despite similar percentages HF, CAD, ASCVD, and stroke in the WS-positive and WS-negative groups, and surprisingly decreased proportions of hypertension and diabetes in the WS-positive group, WS-positive patients had higher age- and sex-adjusted risk of incidental cardiovascular outcomes and mortality, continuing to corroborate the importance of this technique and LVH as independent risk factors for cardiovascular disease. Dissecting the etiological and genetic mechanisms that drive the resulting radiomic and electrical phenotypes of LVH remain an area of active research, which may improve current prognostic understandings of LVH to specific subpopulations and guide more targeted therapy.

## Conclusion

In conclusion, we demonstrate a novel and simplified ECG technique to detect LVH, and measure its performance, along with established LVH techniques, on the largest ECG-TTE linked dataset for this task. The WS technique, which relies on the simple assessment of visually overlapping QRS complexes in the precordial leads, is comparatively easier to remember, faster to perform, and has similar performance to established techniques.

## Data Availability

The datasets presented in this article are not readily available because; Personally identifiable health data that is currently limited to the authors of the article and those covered under the IRB. Requests to access the datasets should be directed to; SS, ssomani@stanford.edu.
