# Supplementary material for: Development and validation of a rapid visual technique for left ventricular hypertrophy detection from the electrocardiogram
Source: Front Cardiovasc Med. 2023 Aug 23;10:1251511. doi: 10.3389/fcvm.2023.1251511 (PMC10499494; doi:10.3389/fcvm.2023.1251511)
Supplement: Supplementary file 1 [file Datasheet1.docx]

**Detailed Methods**

The ECG-LVH dataset was built first by identifying all ECGs performed at Stanford Health Care from March 1, 2006 through May 31, 2018. ECGs were available from commercial software from Philips Healthcare (Andover, MA) in XML files, which were parsed to extract the raw waveform data composed of six limb and six precordial lead recordings of 10 seconds duration sampled at 500Hz. The amplitude of each ECG lead’s waveform was normalized against the calibration wave for that lead to convert the waveform measurement into millimeters (the size of one small box on a standard 12-lead printout). The following filters were sequentially applied to the waveforms: baseline wander removal (to account for breathing artifacts), bandpass filter (to remove excessive variation noise). ECGs with bundle branch blocks (left and right), paced rhythms, or quality control warnings were excluded from this study.

Corresponding resting transthoracic echocardiogram (TTE) reports were obtained, if available, for all patients present in the database. TTE reports were generated and stored on the Philips Xcelera picture archiving and communication system. TTE reports were removed if following variables were missing: LV posterior wall diameter at diastole (LVPWd), LV internal diameter at diastole (LVIDd), interventricular septum diameter at diastole (IVSd), and body surface area. LV mass index (LVMI) for each echocardiogram was calculated using the Devereaux formula for LVH and normalized to the body surface area (BSA).^1^ LVH was defined as LVMI > 95 g/m^2^ in males and LVMI > 115 g/m^2^ in females.

Demographics (age, sex, ethnicity, race) and comorbidities (hypertension, diabetes mellitus, chronic kidney disease by stage, dialysis dependence, heart failure, coronary artery disease, history of prior myocardial infarction, history of prior stroke, and presence of any atherosclerotic cardiovascular disease) were also obtained for these patients using STARR-OMOP^2^, a common data model for accessing Stanford electronic health records. Comorbidities were obtained using the OMOP concept codes listed in Supplemental Table 1. Follow-up mortality and disease data were queried from STARR-OMOP. Cardiovascular mortality was defined as death in the electronic health record falling within thirty days of a condition-record of myocardial infarction, ischemic stroke, intracranial hemorrhage, sudden cardiac death, or hospitalization for heart failure. We also evaluated for the development of myocardial infarction, heart failure, and any cardiovascular disease (defined as the incidence of MI, stroke/hemorrhage, sudden cardiac death, and heart failure hospitalization). A 10-year horizon was chosen; events after this period were censored.

Since all criteria required identification of R- and S-wave amplitudes, we chose to identify QRS complexes from native, non-PVC beats. QRS complexes were identified using the Christov algorithm, given its robustness against premature ventricular contractions (PVCs) relative to the more conventionally used Pan-Tompkins algorithm (**Supplemental Figure** **1**).^3,4^ This algorithm identifies the R-wave based on an expected QRS waveform – with a predominant R-wave and physiologic Q- and S-waves. Lead V6 is used since it is most likely to have this typical waveform shape (e.g. other leads may not have this waveform in the face of severe ventricular hypertrophy or cardiomyopathy). R-wave and S-wave amplitudes were chosen as the maximum positive and maximum negative values, respectively, of the waveform 75 milliseconds before and 75 milliseconds after the location of the identified R-wave. Scores for all criteria in each technique were recorded for each QRS complex for each ECG. An ECG was deemed to contain LVH by that technique if at least half the QRS complexes met the LVH detection threshold for any criteria in that technique.

Technique performance was evaluated in multiple ways. Note that each technique, whose output ultimately is binary (e.g. LVH or no LVH), is composed of multiple criteria, each of which is composed of a continuous value representing the sum of various voltages on the ECG. For example, the Sokolow-Lyon technique is composed of two criteria: S_V1_ + R_V5_ and S_V1_ + R_V6_. Conceptually, we can represent all techniques as sets composed of all individual criterion for that technique:

$$\mathbf{W}_{b_{j}}=\left\{ S_{V_{i,j}}+R_{V_{i+1,j}} | i\in\left[ 1,5 \right] \right\}$$

$$\mathbf{P}_{b_{j}}=\left\{ S_{V_{4,j}}+S_{V_{i},j} | i\in\left[ 1,6 \right] \right\}$$

$$\mathbf{S}_{b_{j}}=\left\{ S_{V_{1}}+R_{V_{i}} | i\in\left[ 5,6 \right] \right\}$$

$$\mathbf{C}_{b_{j}}=\left\{ R_{a_{VL},j}+S_{V_{3}} \right\}$$

Where **W** represents the set of WS criteria, **P** represents the set of Peguero-Lo Presti criteria, **S** represents the set of Sokolow-Lyon criteria, and **C** represents the set of Cornell criterion, all of which contain *c_T_* elements (5 for WS, 6 for Peguero-Lo Presti, 2 for Sokolow-Lyon, and 1 for Cornell); *b_j_* represents a specific heartbeat *j* for a ECG recording, $S_{V_{i,j}}$ represents the S-wave corresponding to lead *V_i_* or lead *aVL* for heartbeat *j*, $R_{V_{i,j}}$ represents the S-wave corresponding to lead *V_i_* or lead *aVL* for heartbeat *j*, and *g* represents the sex of the patient. For an explicit example, the Sokolow-Lyon set **S**, for a given heartbeat *b_j_*, will be a set containing two elements:

$$S_{b_{j}}=\{S_{V_{1}}+R_{V_{5}}, S_{V_{1}}+R_{V_{6}}\}$$

We then enforce that LVH be detected for a technique *T* once at least half of all heartbeats meet the threshold for LVH as defined by the following:

$${LVH}_{\mathbf{T}_{\mathbf{a}}}=\left\{ \frac{1}{N}\sum_{j=1}^{N} {(\mathbf{T}_{\mathbf{a}}}_{b_{j}}+\left( 1-S_{k} \right)*g_{T})\geq t_{T} \right\}\geq0.5$$

$$\mathrm{where} \mathbf{T}\in\left\{ \mathbf{W}, \mathbf{P}, \mathbf{S}, \mathbf{C} \right\}$$

Where *LVH* represents LVH as detected by that technique T_a_ in an ECG containing *N* heartbeats, *g_T_* represents the sex-adjustment (5 for Peguero-Lo Presti, 8 for Sokolow, and 0 for WS and Cornell), *t_T_* represents the threshold for that technique as listed in Supplementary Table 2, and *S_k_* represents the sex (male = 1, female = 0) of patient *k* to whom the current ECG belongs. Classification metrics are performed from this binary representation of LVH.

Given that the pre-existing thresholds *t_T_* for declaring LVH may be suboptimal, subsequent threshold-independent analysis of techniques is performed by treating the output of any given technique as a continuous feature. To reduce the plurality of all criteria in an LVH detection technique across all detected heartbeats, we choose the maximum of the median of all criteria values across all heartbeats (MMCV):

$$M_{T}= \max_{k \in[1, c_{T}]} \left\{ \underset{j\in\left[ 1,N \right]}{\mathrm{median}} \mathbf{T}_{b_{j}} \right\}+\left( 1-S_{k} \right)*g_{T})$$

where *k* represents each criterion in the LVH detection technique T. Taking the median value of each criterion value allows a representative value for that criterion, as opposed to other statistical measures (mean) that could be confounded by a PVC. Taking the max of this set of values fits within the context of how these techniques are designed (e.g. detecting LVH when any criteria are satisfied).

Receiver-operator and precision-recall curves are generated by using MMCV as the independent variable and the presence of LVH as the dependent variable. The relationship between the MMCV and a more continuous representation of the degree of LVH, the LVMI, is then detected using a simple linear regression model to the and measuring the resulting Pearson statistic using the scikit-learn LinearRegression model.

Sensitivity analyses were performed by assessing all relevant classification metrics after varying the sex-adjustment (*g_W_*) in the LVH equation (*LVH_TW_*) for the WS technique above. Other visual criteria, such as lateral lead T-wave inversions, lateral lead ST-segment depressions, and left atrial enlargement, were extracted from the interpretation statements on the ECG (**Supplementary Table 7**).

Survival analyses were conducted by using age, sex, and either the MMCV (*M_T_*) or binary LVH detection result for the technique (*LVH_Ta_*) as input features to standard Cox proportional hazards models using the lifelines package in Python.^5^ All confidence intervals are 95% Cox proportional hazards confidence intervals. A ten-year time horizon was enforced for the dataset; all patients without events after 10-years were censored at that time mark. Model fit was evaluated using the Harrell’s C-statistic.^6^ Statistical significance when LVH was or was not detected by *LVH_Ta_* and thresholded at 50, 75, 85, 95, and 99 percentile values of *M_T_* were evaluated using the Wald test statistic.

Relevant code used in the development of this work can be found at www.github.com/sssomani/
LVH_ECG.

**Supplementary Figure 1.** Performance of Pan-Tompkins vs. Christov Algorithms in Avoiding PVC Detection. Example ECGs from two patients (left, right) and all heartbeats detected using the Pan-Tompkins (top) and Christov (bottom) algorithms.


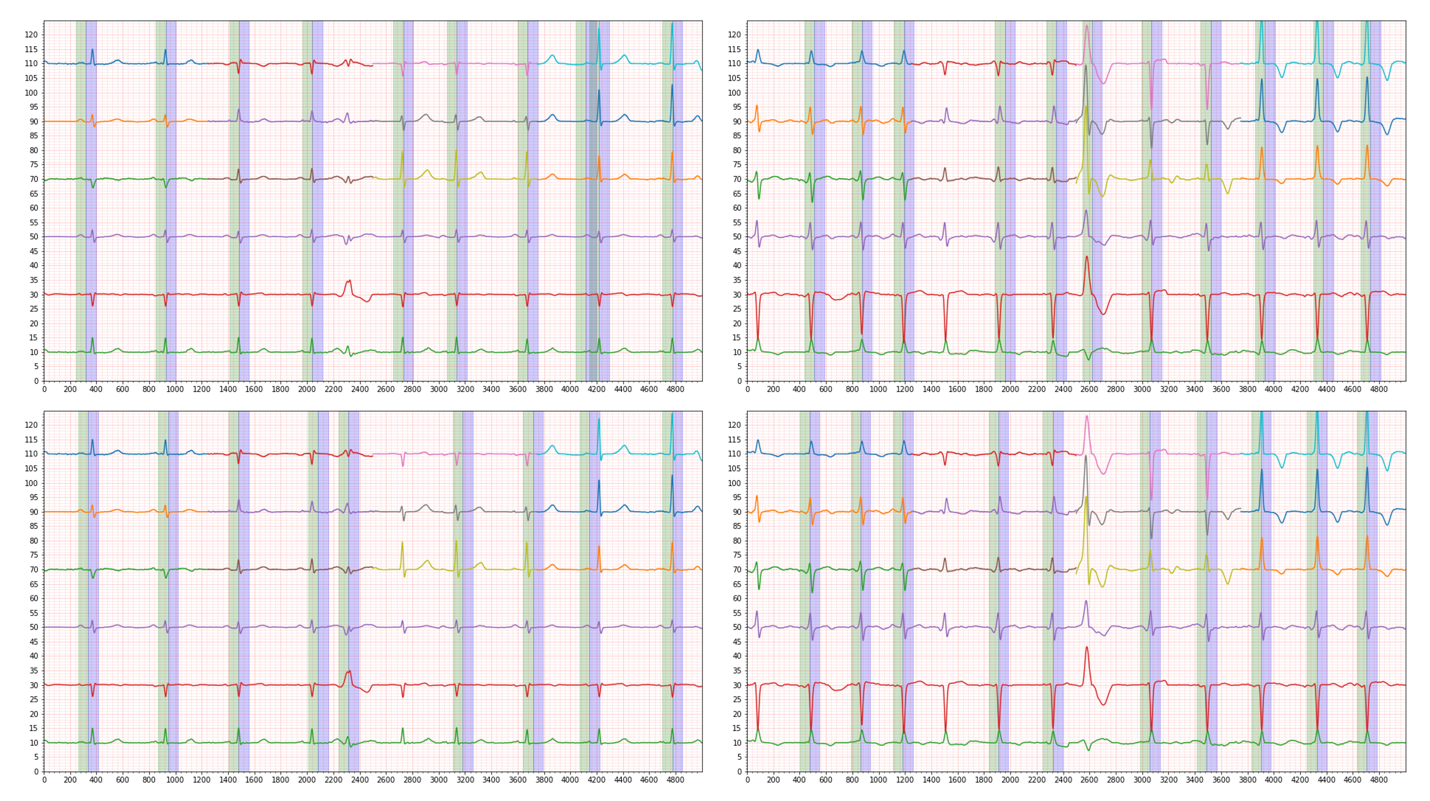


**Supplementary Figure 2**. Sensitivity analysis for sex-based threshold-adjustment for the WS technique.


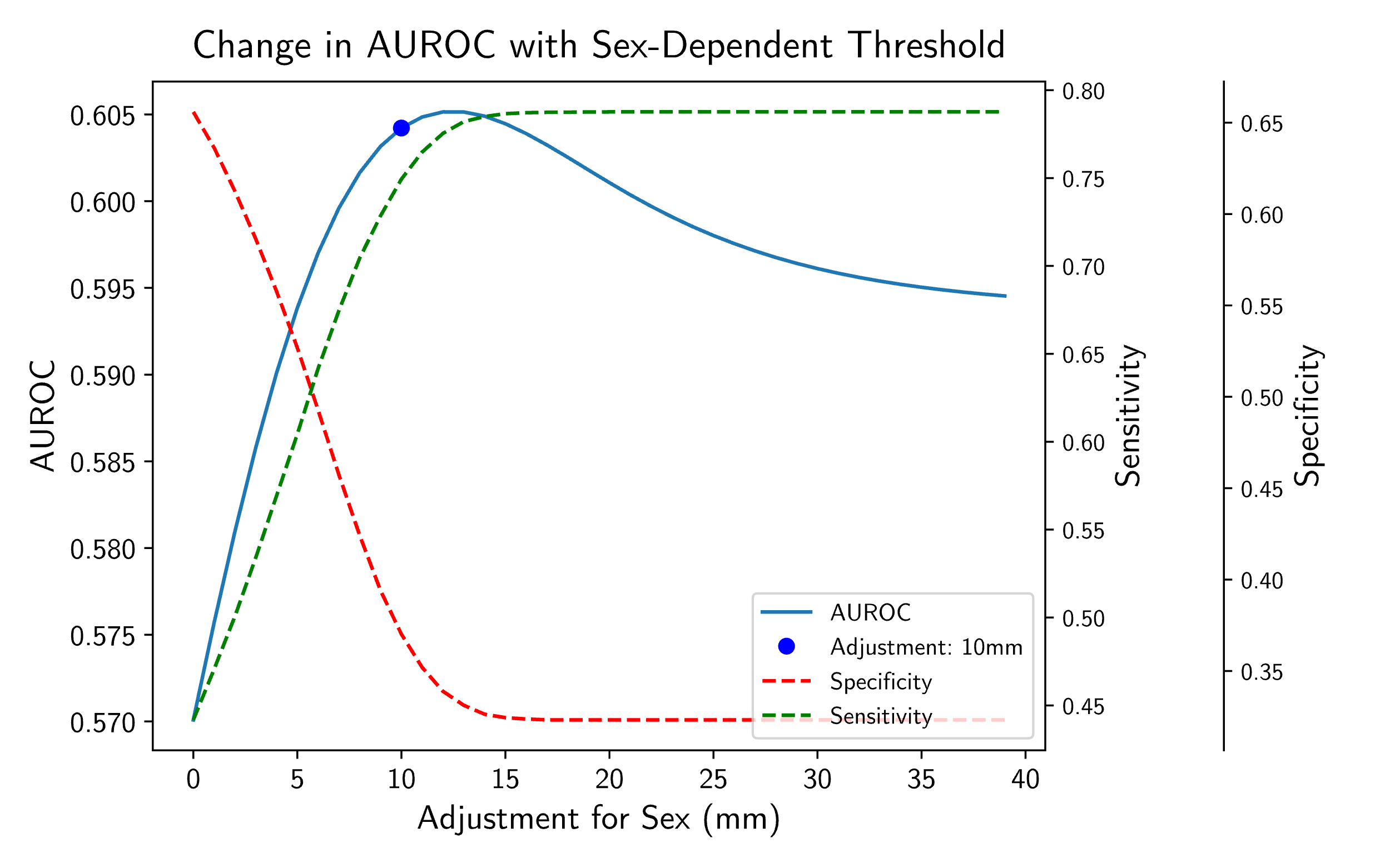


**Supplementary Figure 3**. Association of LVH Technique with LVMI. Scatter plots of MMCV values from ECG with corresponding LVMI from TTE for all techniques. Abbreviations: LVMI: LV mass index, MMCV: maximum across the median of all criterion values across all heartbeats.


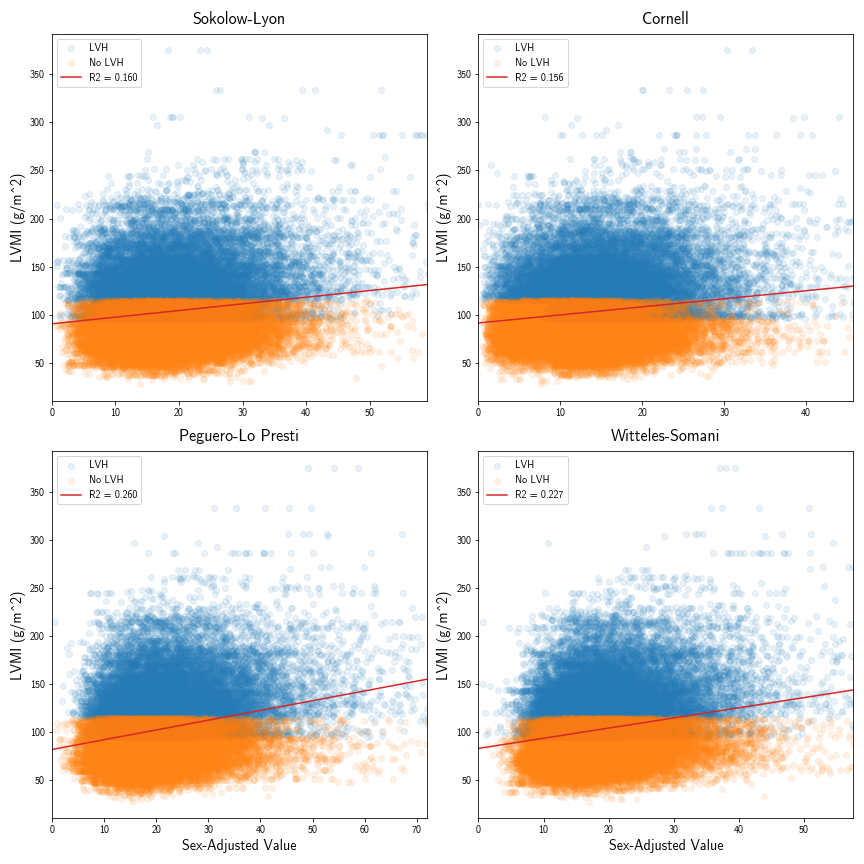


**Supplementary Figure 4.** Association with Cardiovascular Outcomes. Survival curves of incidental myocardial infarction, cardiovascular disease, heart failure, and mortality, with associated hazard ratios and P-values using the MMCV by the Witteles-Somani technique. Curves with thresholding the MMCV at the 50, 75, 85, 95, 99 percentile thresholds for LVH are also shown to assess covariance.

**
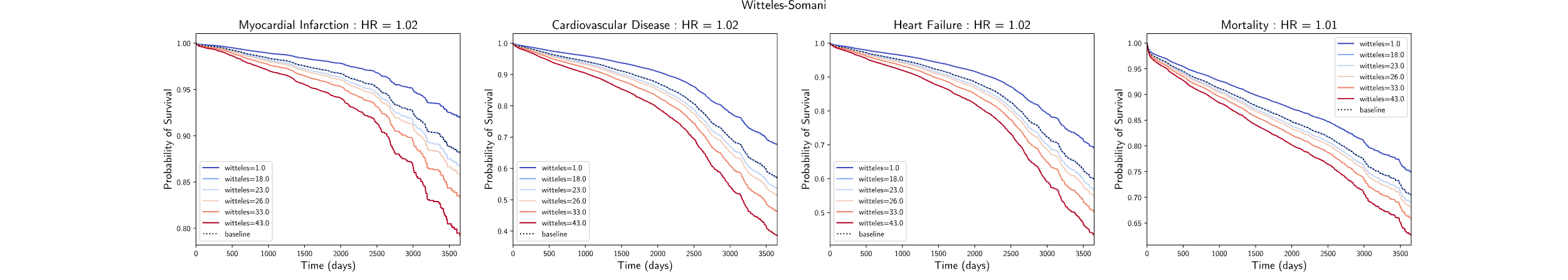
**

**Supplementary Figure 5.** Association with Cardiovascular Outcomes. Survival curves of incidental myocardial infarction, cardiovascular disease, heart failure, and mortality, with associated hazard ratios and P-values using the presence or absence of LVH by (top) and the MMCV by the Cornell technique (bottom). Curves with thresholding the MMCV at the 50, 75, 85, 95, 99 percentile thresholds for LVH are also shown to assess covariance.


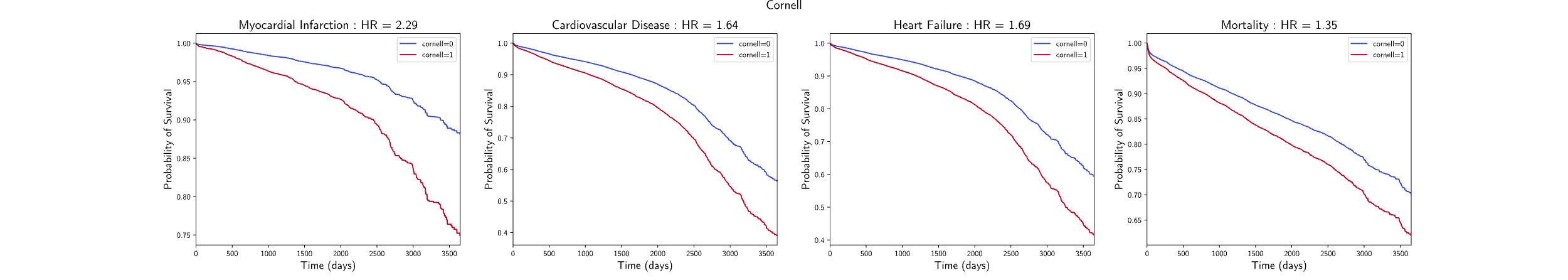


**
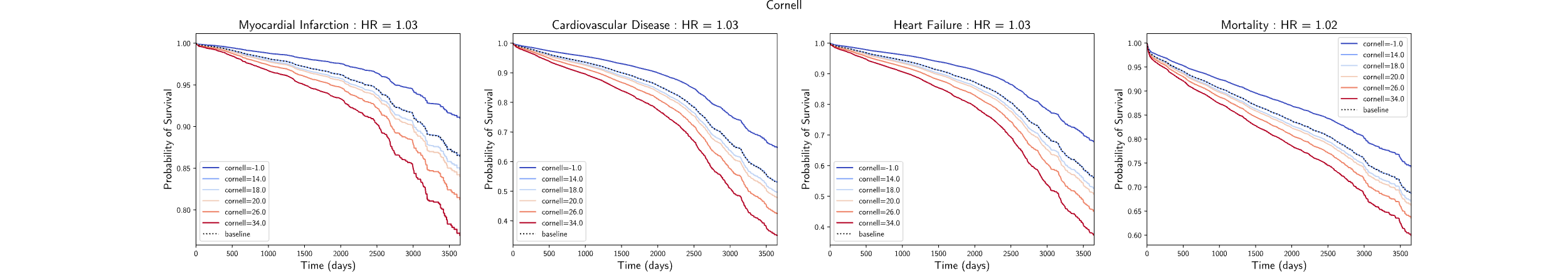
Supplementary Figure 6.** Association with Cardiovascular Outcomes. Survival curves of incidental myocardial infarction, cardiovascular disease, heart failure, and mortality, with associated hazard ratios and P-values using the presence or absence of LVH by (top) and the MMCV by the Sokolow-Lyon technique (bottom). Curves with thresholding the MMCV at the 50, 75, 85, 95, 99 percentile thresholds for LVH are also shown to assess covariance.

**
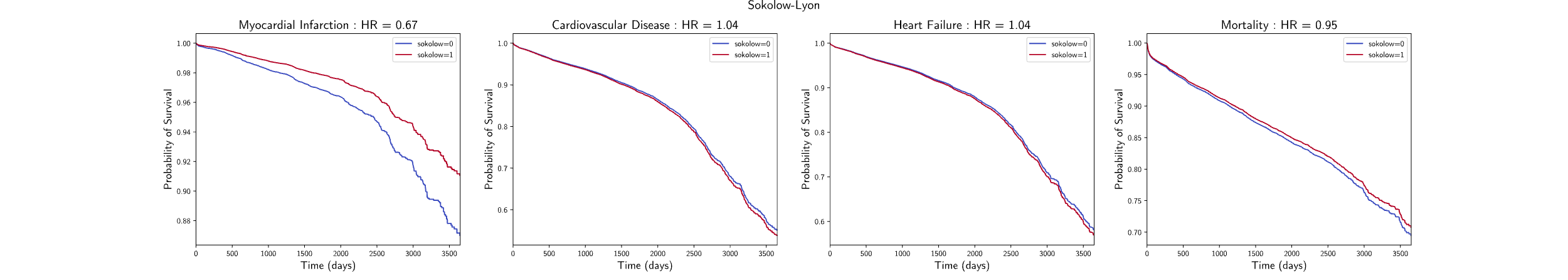
**

**
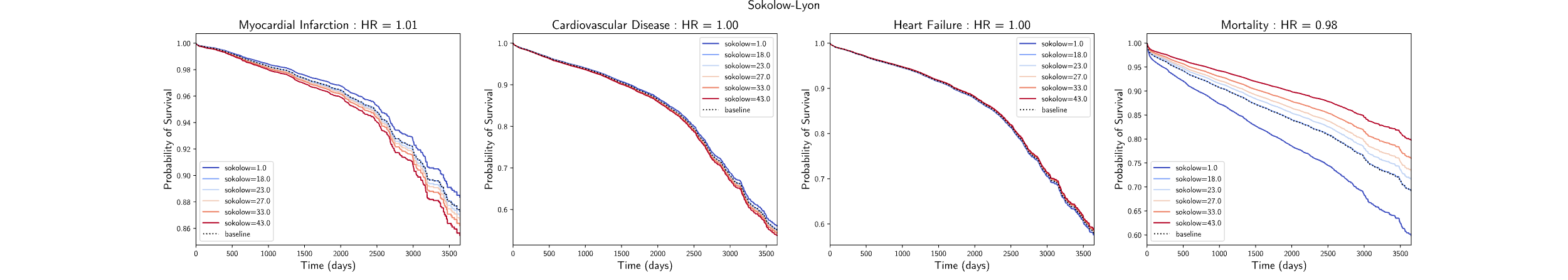
**

**Supplementary Figure 7.** Association with Cardiovascular Outcomes. Survival curves of incidental myocardial infarction, cardiovascular disease, heart failure, and mortality, with associated hazard ratios and P-values using the presence or absence of LVH by (top) and the MMCV by the Peguero-Lo Presti technique (bottom). Curves with thresholding the MMCV at the 50, 75, 85, 95, 99 percentile thresholds for LVH are also shown to assess covariance.


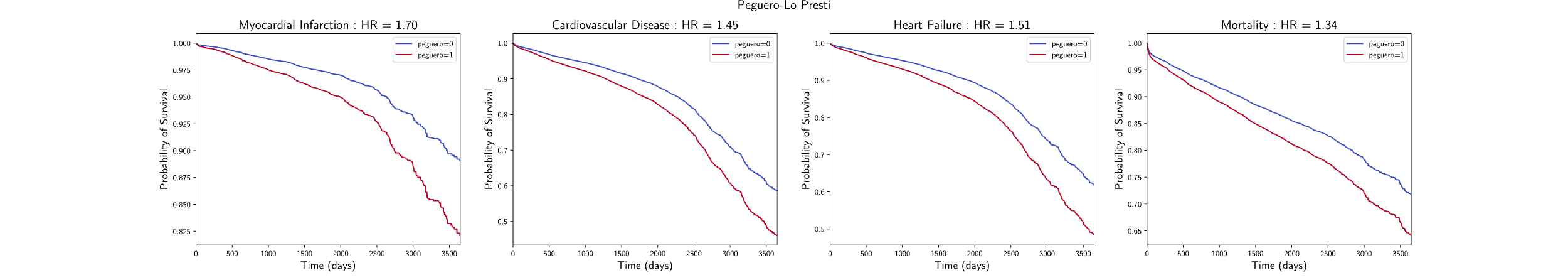


**
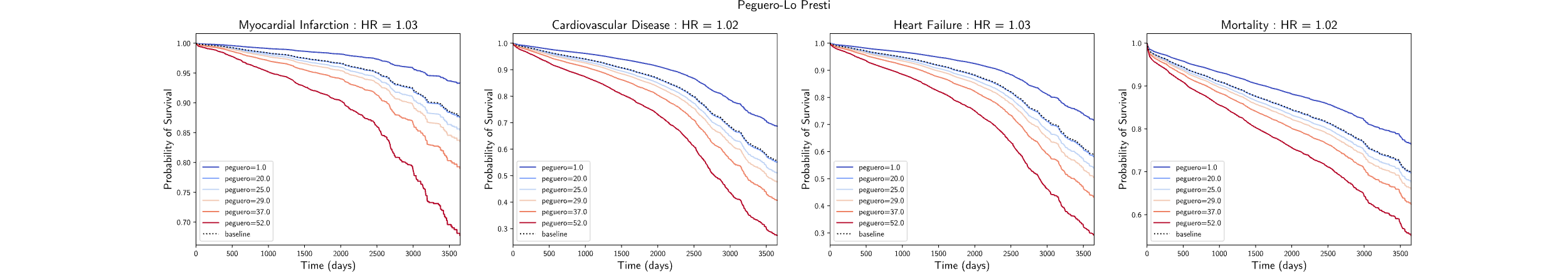
**

**Supplementary Figure 8**. Association with Cardiovascular Outcomes. Survival curves of incidental myocardial infarction, cardiovascular disease, heart failure, and mortality, with associated hazard ratios and P-values using the presence or absence of LVH by (top) TTE and the LVMI (bottom). Curves with thresholding the LVMI at the 50, 75, 85, 95, 99 percentile thresholds for LVH are also shown to assess covariance.


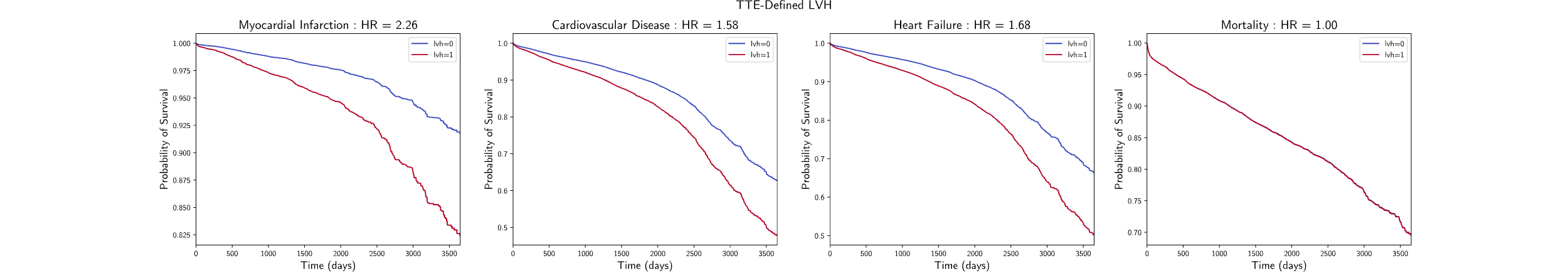


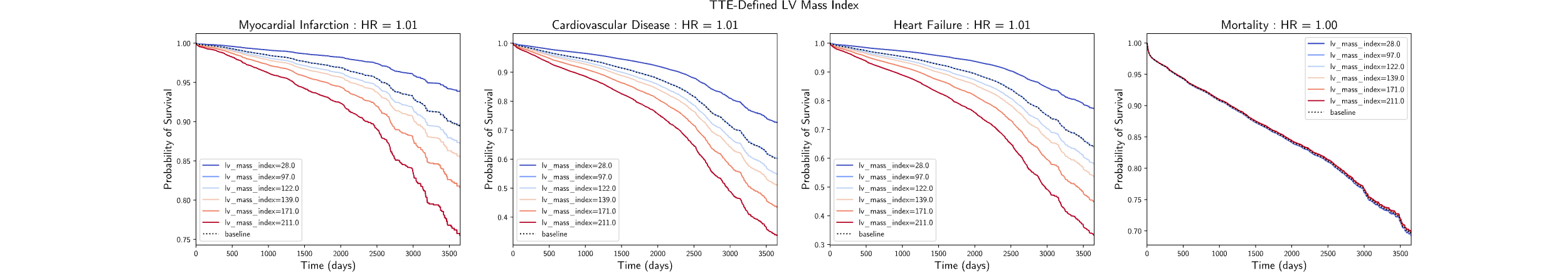


**Supplementary Table 1: OMOP concept codes for conditions and lab values**

| **Concept Name** | **Included concept codes** | **Excluded concept codes** |
| --- | --- | --- |
| Myocardial infarction | 4329847 | 314666 (old myocardial infarction) |
| Ischemic stroke and intracranial hemorrhage | 372924, 375557, 376713, 443454, 441874, 439847, 432923 |  |
| Sudden cardiac death | 4048809, 321042, 442289, 4317150, 4132309 | 437894 (ventricular fibrillation) |
| Heart failure | 316139 | 315295 (congestive rheumatic heart failure) |
| Atrial fibrillation | 313217 |  |
| Diabetes | 443735, 443767, 192279, 443732, 376065, 443729, 201826, 4225656, 4227210, 435216, 37016355, 4228112, 4224254, 200687, 201254, 201531, 4295011 |  |

**Supplementary Table 2:** Thresholds for Various LVH Techniques

| **Technique** | **Threshold** |
| --- | --- |
| Sokolow-Lyon | 35 |
| Cornell | 28 |
| Peguero-Lo Presti | 28 |
| Witteles-Somani | 20 |

**Supplementary Table 3**. Measures of LVH Detection Technique Performance.

| **Criteria** | **Accuracy** | **F1-Score** | **PPV** | **NPV** | **Sensitivity** | **Specificity** |
| --- | --- | --- | --- | --- | --- | --- |
| Sokolow-Lyon | 0.602 | 0.102 | 0.602 | 0.602 | 0.056 | **0.975** |
| Cornell | 0.612 | 0.180 | **0.631** | 0.611 | 0.105 | 0.958 |
| Peguero Lo-Presti | **0.618** | 0.413 | 0.548 | **0.640** | 0.331 | 0.814 |
| Witteles-Somani | 0.569 | **0.454** | 0.467 | 0.632 | **0.442** | 0.656 |

**Supplementary Table 4**. Impact of Combining LVH Detection Techniques. WS: Witteles-Somani, C: Cornell, SL: Sokolow-Lyon, P: Peguero-Lo Presti.

| **Technique** | **Accuracy** | **Sensitivity** | **Specificity** | **PPV** | **NPV** |
| --- | --- | --- | --- | --- | --- |
| **WS or SL** | 0.569 | 0.443 | 0.655 | 0.467 | 0.633 |
| **WS and SL** | 0.602 | 0.055 | 0.976 | 0.606 | 0.602 |
| **WS or C** | 0.569 | 0.444 | 0.655 | 0.467 | 0.633 |
| **WS and C** | 0.612 | 0.103 | 0.959 | 0.633 | 0.610 |
| **WS or P** | 0.572 | 0.506 | 0.617 | 0.474 | 0.647 |
| **WS and P** | 0.615 | 0.267 | 0.853 | 0.553 | 0.630 |
| **SL or C** | 0.615 | 0.142 | 0.937 | 0.607 | 0.615 |
| **SL and C** | 0.599 | 0.018 | 0.996 | 0.750 | 0.598 |
| **SL or P** | 0.617 | 0.346 | 0.802 | 0.544 | 0.642 |
| **SL and P** | 0.603 | 0.040 | 0.987 | 0.678 | 0.601 |
| **C or P** | 0.618 | 0.332 | 0.813 | 0.548 | 0.641 |
| **C and P** | 0.612 | 0.104 | 0.959 | 0.633 | 0.611 |
| **WS or SL or C** | 0.569 | 0.444 | 0.654 | 0.467 | 0.633 |
| **WS and SL and C** | 0.599 | 0.018 | 0.996 | 0.755 | 0.598 |
| **WS or SL or P** | 0.572 | 0.506 | 0.616 | 0.474 | 0.647 |
| **WS and SL and P** | 0.603 | 0.040 | 0.987 | 0.681 | 0.601 |
| **WS or C or P** | 0.572 | 0.506 | 0.617 | 0.474 | 0.646 |
| **WS and C and P** | 0.612 | 0.102 | 0.960 | 0.634 | 0.610 |
| **SL or C or P** | 0.617 | 0.347 | 0.801 | 0.543 | 0.642 |
| **SL and C and P** | 0.599 | 0.018 | 0.996 | 0.753 | 0.598 |
| **WS or SL or C or P** | 0.572 | 0.506 | 0.616 | 0.474 | 0.647 |
| **WS and SL and C and P** | 0.599 | 0.018 | 0.996 | 0.756 | 0.598 |

**Supplementary Table 5**. Impact of Visual ECG Signs on WS Performance. LAE: left atrial enlargement, STD: ST-segment depressions in at least the lateral leads, TWI: T-wave inversions in at least the lateral leads

| **Technique** | **Accuracy** | **Sensitivity** | **Specificity** | **PPV** | **NPV** |
| --- | --- | --- | --- | --- | --- |
| **WS** | 0.57 | 0.44 | 0.66 | 0.47 | 0.63 |
| **LAE** | 0.61 | 0.05 | 0.98 | 0.67 | 0.60 |
| **TWI** | 0.59 | 0.01 | 0.99 | 0.41 | 0.59 |
| **STD** | 0.59 | 0.01 | 1.00 | 0.48 | 0.59 |
| **WS + LAE** | 0.60 | 0.03 | 0.99 | 0.74 | 0.60 |
| **WS + STD** | 0.59 | 0.00 | 1.00 | 0.53 | 0.59 |
| **WS + TWI** | 0.59 | 0.01 | 0.99 | 0.43 | 0.59 |
| **WS + LAE + TWI** | 0.59 | 0.00 | 1.00 | 0.00 | 0.59 |
| **WS + LAE + STD** | 0.59 | 0.00 | 1.00 | 0.00 | 0.59 |
| **WS + STD + TWI** | 0.59 | 0.00 | 1.00 | 1.00 | 0.59 |
| **WS + LAE + STD + TWI** | 0.59 | 0.00 | 1.00 | 0.00 | 0.59 |
| **WS or LAE** | 0.57 | 0.46 | 0.65 | 0.47 | 0.64 |
| **WS or STD** | 0.57 | 0.44 | 0.65 | 0.47 | 0.63 |
| **WS or TWI** | 0.57 | 0.45 | 0.65 | 0.47 | 0.63 |
| **WS or LAE or STD or TWI** | 0.57 | 0.47 | 0.63 | 0.47 | 0.64 |

**Supplementary Table 6:** Association with Cardiovascular Outcomes for LVH Techniques.

|  |  | **MI** | | | **CVD** | | | **HF** | | | **Mortality** | | |
| --- | --- | --- | --- | --- | --- | --- | --- | --- | --- | --- | --- | --- | --- |
| **Input Type** | **Technique** | **Hazard Ratio**  **[95% CI]** | **P-Value** | **C-Index** | **Hazard Ratio**  **[95% CI]** | **P-Value** | **C-Index** | **Hazard Ratio**  **[95% CI]** | **P-Value** | **C-Index** | **Hazard Ratio**  **[95% CI]** | **P-Value** | **C-Index** |
| Binary | Sokolow-Lyon | 1.04 [0.90, 1.20] | 6.0E-01 | 0.55 | 1.04 [0.89, 1.20] | 6.3E-01 | 0.57 | 0.67 [0.48, 0.94] | 2.1E-02 | 0.55 | 0.95 [0.83, 1.08] | 4.4E-01 | 0.55 |
|  | Cornell | 1.64 [1.48, 1.81] | 2.4E-22 | 0.56 | 1.69 [1.53, 1.87] | 2.2E-25 | 0.58 | 2.29 [1.96, 2.69] | 7.5E-25 | 0.59 | 1.35 [1.24, 1.48] | 1.9E-11 | 0.55 |
|  | Peguero-Lo Presti | 1.45 [1.36, 1.54] | 3.8E-32 | 0.57 | 1.51 [1.42, 1.61] | 9.9E-39 | 0.58 | 1.70 [1.52, 1.90] | 2.1E-20 | 0.59 | 1.34 [1.27, 1.41] | 1.9E-25 | 0.56 |
|  | Witteles-Somani | 1.29 [1.22, 1.36] | 1.6E-18 | 0.56 | 1.24 [1.17, 1.32] | 1.2E-13 | 0.57 | 1.60 [1.44, 1.78] | 1.0E-17 | 0.58 | 1.18 [1.12, 1.24] | 8.5E-11 | 0.55 |
| MMCV | Sokolow-Lyon | 1.00 [1.00, 1.01] | 3.6E-01 | 0.55 | 1.00 [1.00, 1.00] | 6.4E-01 | 0.57 | 1.01 [1.00, 1.01] | 9.1E-02 | 0.56 | 0.98 [0.98, 0.98] | 1.9E-30 | 0.57 |
|  | Cornell | 1.03 [1.02, 1.03] | 1.8E-65 | 0.57 | 1.03 [1.02, 1.03] | 1.6E-81 | 0.58 | 1.03 [1.03, 1.03] | 9.7E-58 | 0.60 | 1.02 [1.01, 1.02] | 2.9E-18 | 0.55 |
|  | Peguero-Lo Presti | 1.02 [1.02, 1.03] | 7.9E-63 | 0.58 | 1.03 [1.02, 1.03] | 5.9E-74 | 0.59 | 1.03 [1.03, 1.04] | 8.7E-50 | 0.61 | 1.02 [1.01, 1.02] | 1.1E-35 | 0.56 |
|  | Witteles-Somani | 1.02 [1.02, 1.03] | 3.2E-29 | 0.57 | 1.02 [1.02, 1.02] | 5.1E-24 | 0.58 | 1.02 [1.02, 1.03] | 3.4E-43 | 0.60 | 1.01 [1.01, 1.01] | 3.8E-14 | 0.55 |

**Supplementary Table 7:** Interpretation Statements in Other Visual ECG Signs

| **ECG Sign** | **Interpretation Statements** |
| --- | --- |
| **T-wave Inversions** | 'Abnormal T, consider ischemia, lateral leads',  'Abnormal T, consider ischemia, ant-lat leads',  'Abnormal T, consider ischemia, diffuse leads',  'Abnormal T, consider ischemia, widespread',  'Abnormal T, consider ischemia, inferolateral',  'Abnormal T, consider ischemia, anterolateral leads' |
| **ST-segment Depressions** | 'Minimal ST depression, lateral leads',  'Minimal ST depression, anterolateral leads',  'Minimal ST depression, diffuse leads',  'Nonspecific ST depression, lateral leads',  'Nonspecific ST depression, ant-lat leads',  'Nonspecific ST depression, diffuse leads',  'ST depression, consider ischemia, lat leads',  'ST depression, consider ischemia, ant-lat lds',  'ST depression, consider ischemia, diffuse lds' |
| **Left Atrial Enlargement** | 'Probably left atrial enlargement',  'Left atrial enlargement’,  'Biatrial enlargement', |

**References**

1. Devereux RB, Alonso DR, Lutas EM, et al. Echocardiographic assessment of left ventricular hypertrophy: comparison to necropsy findings. *Am J Cardiol*. 1986;57(6):450-458.

2. Datta S, Posada J, Olson G, et al. A new paradigm for accelerating clinical data science at Stanford Medicine. *arXiv [csCY]*. Published online March 17, 2020. http://arxiv.org/abs/2003.10534

3. Pan J, Tompkins WJ. A real-time QRS detection algorithm. *IEEE Trans Biomed Eng*. 1985;32(3):230-236.

4. Christov II. Real time electrocardiogram QRS detection using combined adaptive threshold. *Biomed Eng Online*. 2004;3(1):28.

5. Davidson-Pilon C. lifelines: survival analysis in Python. *J Open Source Softw*. 2019;4(40):1317.

6. Harrell FE Jr, Califf RM, Pryor DB, Lee KL, Rosati RA. Evaluating the yield of medical tests. *JAMA*. 1982;247(18):2543-2546.
